# Three-dimensional kinematic gait signatures of idiopathic normal pressure hydrocephalus: a biomechanical framework toward objective diagnosis

**DOI:** 10.1186/s12987-026-00813-6

**Published:** 2026-05-22

**Authors:** Richard Mills, Tobias Langheinrich, Liis Uiga, Katherine A. J. Daniels, Sean Maudsley-Barton, Mariam Riaz, Cliff Chen, Owen Thomas, Claire Atherton, Neil D. Reeves, Mats Tullberg

**Affiliations:** 1 Department of Sport and Exercise Sciences, Manchester Metropolitan University, Manchester, UK; 2 Manchester Centre for Clinical Neurosciences, Northern Care Alliance NHS Foundation Trust, Manchester, UK; 3 Division of Psychology, Communication and Human Neuroscience, University of Manchester, Manchester, UK; 4 Department of Computing and Mathematics, Manchester Metropolitan University, Manchester, UK; 5 Division of Informatics, Imaging and Data Sciences, University of Manchester, Manchester, UK; 6 Medical School, Faculty of Health and Medicine, Lancaster University, Lancaster, UK; 7 Hydrocephalus Research Unit, Department of Clinical Neuroscience, Institute of Neuroscience and Physiology, Sahlgrenska Academy, University of Gothenburg, Gothenburg, Sweden; 8 Department of Neurology, Adult Hydrocephalus Care Program, Sahlgrenska University Hospital, Blå stråket 5, Gothenburg, SE-41345 Sweden

**Keywords:** Idiopathic normal pressure hydrocephalus, iNPH, Gait, 3D motion analysis, Kinematics

## Abstract

**Background:**

Idiopathic Normal Pressure Hydrocephalus (iNPH) is a leading cause of reversible gait disturbance in older adults, yet diagnosis and treatment selection remain limited by low-sensitivity clinical assessments. Conventional tests such as the 10-metre walk and Timed Up-and-Go capture overall performance but overlook the multidimensional, joint-level alterations that characterise iNPH gait. This study aimed to deliver the first comprehensive, three-dimensional (3D) biomechanical characterisation of iNPH gait to inform more objective diagnostic frameworks.

**Methods:**

Twenty-three participants with clinically diagnosed iNPH and eighteen age-matched healthy controls underwent baseline gait analysis (iNPH: pre tap test) consisting of overground walking using a 3D optoelectronic motion capture system with integrated force plates. Spatiotemporal parameters were compared using independent-samples *t*-tests, while continuous kinematic (i.e., joint angle) waveforms for the pelvis, hip, knee, and ankle were analysed using one-dimensional statistical parametric mapping (SPM). A binary logistic regression model was developed to classify participants as iNPH or healthy control.

**Results:**

Compared with controls, individuals with iNPH demonstrated markedly slower gait speed (–60%), shorter stride length (–52%), and greater stride width (+ 64%), each with large effect sizes (1.5–3.3; *p* < .001). SPM revealed multi-joint, phase-specific impairments in sagittal and frontal plane kinematics, including reduced pelvic obliquity, diminished hip flexion/extension and abduction, attenuated knee flexion during swing, and blunted ankle plantarflexion at push-off (*p* < .05). These findings indicate impaired supraspinal coordination consistent with frontal-subcortical motor network dysfunction. Further, the statistical modelling achieved excellent discrimination between patients with iNPH and healthy controls. Due to short-stepped iNPH gait causing multiple consecutive steps on a single force plate, kinetics (i.e., ground reaction forces; joint moments) could not be analysed.

**Conclusions:**

This study suggests the first detailed biomechanical signatures of gait in iNPH, identifying spatiotemporal and joint-specific alterations that together define a central coordination deficit. Beyond confirming known gait slowing, these results delineate kinematic markers with translational potential as digital biomarkers for diagnosis and postoperative monitoring. By advancing gait analysis from simple bedside tests to detailed quantification, 3D motion capture offers a rigorous, reproducible framework that may improve diagnostic sensitivity in patients with iNPH. However, larger future studies should include clinically more heterogeneous cohorts and incorporate relevant disease mimics (e.g., Parkinson’s disease, progressive supranuclear palsy) to determine differentiation capabilities of the predictive modelling.

**Supplementary Information:**

The online version contains supplementary material available at 10.1186/s12987-026-00813-6.

## Background

Idiopathic normal pressure hydrocephalus (iNPH) is the most common form of adult hydrocephalus with an estimated prevalence of 3% among individuals over 65 years and 6–9% among individuals over 80 years old [1–4]. The characteristic clinical triad includes impaired gait and balance, cognitive decline, and urinary incontinence [5, 6], symptoms that are significantly and rapidly reversed by shunt surgery in up to 80% of patients [7–10].

iNPH remains both underdiagnosed and undertreated, with only approximately 20–40% of affected individuals ultimately receiving treatment, shunt surgery [3]. One main reason for this is the lack of sufficiently sensitive and reliable diagnostic methods to identify iNPH early. The diagnostic process requires recognition of typical clinical symptoms, identification of specific neuroimaging features, and exclusion of mimics, mainly neurodegenerative and cerebrovascular diseases, as well as age-related impairments.

Assessment of gait disturbance is central for clinical diagnosis of iNPH and evaluation of treatment outcomes [11]. Currently, gait assessment for this condition is primarily based on visual observation, focussing on features of neurological gait disorders such as broad-based, short-stepped, and shuffling gait, as well as turning difficulties (often described as a “higher-level gait disorder”), and is considered in conjunction with findings on neurological examination of cranial nerves and limbs. These characteristics are also often considered alongside simple quantitative clinical performance functional tests, such as the 10-meter walking test (10MWT) or the Timed-up and Go (TUG) test. Though widely used, visual assessments of gait are subjective and can be prone to high inter-rater variance, and the 10MWT and TUG are generic mobility measures that, while capturing gait outcomes (i.e., average walking speed), do not provide detailed mechanical characteristics of gait. As a result, these approaches have limited sensitivity for detecting subtle preoperative symptoms, supporting differential diagnosis.

Visual assessments are often supported through the use of 2-dimensional video recordings, enabling repeat analysis by different raters but with no universally accepted, standardized rating system. Using these techniques, previous studies have identified clear impairments in iNPH gait rooted in temporally-based metrics, including slower gait speed, shorter step lengths, and reduced cadence [12–15]. More detailed gait metrics describing quality of movement are available through three-dimensional motion capture by recording multi-planar joint characteristics and temporal events with high precision, providing a more objective quantification of subtle deficits in gait. These features – primarily joint angles and angular velocities (‘kinematics’, describing movement) and joint loading, including the forces and moments acting on the body (‘kinetics’, describing the mechanical causes of movement) – are commonly reported in biomechanical evaluations of gait [16]. In clinical populations, these detailed features (obtained through 3D motion capture gait analysis) have been shown to contribute to clinical decision making and treatment outcome evaluations by understanding the biomechanics acting at the joint level [17]. Further, when combined with machine learning techniques, features from 3D gait analysis demonstrate promising utility as a diagnostic tool [ 18].

While three-dimensional motion capture offers a sensitive method of gait analysis, it has to date been underutilised for the characterisation of gait in patients with iNPH. There is currently a lack of information regarding joint kinematics and kinetics in iNPH gait – features which may prove helpful in aiding iNPH diagnosis. Few studies have used this technique for identification and monitoring of people with iNPH, (cf. [19]) and none has to our knowledge reported full gait analyses which include joint kinetics and kinematics, instead only reporting simple outcome measures that could be obtained through more rudimentary techniques. Therefore, there is a need for a sensitive gait analysis method to precisely characterise the biomechanical signatures of iNPH gait.

The aim of this study is to provide the first comprehensive characterisation of gait impairment including both joint kinetics and kinematics in people with iNPH compared to healthy controls. We hypothesized that compared to age-matched healthy controls, patients with iNPH would exhibit lower gait speed, increased step width, decreased step length, lower cadence, and altered joint kinematics and kinetics. We also hypothesised that joint kinematic and kinetic characteristics could be used to differentiate iNPH patients from healthy controls with high sensitivity and specificity using logistic regression analysis.

## Methods

### Study cohort, recruitment and consent

People with iNPH (“iNPH” group; *N* = 25) were recruited from the regional specialist NPH clinic at The Manchester Centre for Clinical Neurosciences at Salford Royal Hospital, Northern Care Alliance NHS Foundation Trust, UK, by a senior neurologist with specialist interest in cognitive neurodegenerative disease (TL). Healthy controls (“HC” group; *N* = 19) were recruited through local networks, including University for the 3rd Age, Research for the Future, and carers of patients.

Inclusion criteria for the iNPH group were (i) diagnosis of iNPH according to international guidelines [28], and (ii) capable of walking independently for a minimum of 20 consecutive steps (use of walking aids was allowed to align with the real-life experiences of many individuals with this condition). Exclusion criteria were (i) other confirmed medical or surgical conditions better explaining patients’ symptoms or co-existing conditions with significant impact on gait and/or balance (e.g., Parkinson’s disease, significant osteoarthritis, amputation of lower limb/appendages, musculoskeletal injury, recent lower-limb surgeries, etc.), (ii) other cause of hydrocephalus; previous surgical procedures for hydrocephalus, (iii) patients on specific medications (e.g., centrally acting) better explaining or with significant impact on patients’ symptoms, (iv) unable to speak and comprehend written and/or verbal English, and (v) unwilling or unable to comprehend informed consent. The inclusion criteria for HC were (i) consenting males and females, matched for age to the iNPH group, and (ii) able to walk unaided for at least 20 consecutive steps. The exclusion criteria were the same as those for iNPH patients.

Eligible people with iNPH were approached by the consultant (TL), explained the study and provided with a study participant information sheet. HC were sent an electronic version of the participant information sheet upon first contact. All participants had at least 24 h to decide whether they wished to participate and were given the opportunity to visit the gait laboratory before their first assessment. Written informed consent was obtained by a member of the study team prior to the gait analysis assessment. Study ethical approval was granted through the NHS Health Research Authority (IRAS project ID 302102).

Both groups were invited to the gait laboratory at Manchester Metropolitan University and underwent the same laboratory-based testing protocols to assess their gait and balance; only the iNPH group received clinical assessments and 3D gait analysis was carried out before patients underwent tap test to assess their baseline state.

### Standard clinical assessments

As part of patients’ routine clinical work-up, standard clinical assessments administered included: (a) mobility and bladder symptoms scored with the Gothenburg iNPH scale [20]. A score of 0 represents maximum symptom burden and 100 equals the performance of a healthy 70-year-old; (b) the modified Rankin Scale (mRS) [21] to assess functional disability and dependence, 0 representing normality, 6 death; (c) cognitive profiles obtained by a senior neuropsychologist with specialist interest in differential diagnostic assessments of patients with dementing disorders (CC); (d) the Addenbrooke’s cognitive examination III [22] to score global cognition, 100/100 representing flawless performance; (e) 3T MRI scan of the brain rated for Evans’ index [23] and iNPH Radscale [24], and disproportionally enlarged subarachnoid space hydrocephalus (DESH) [25] by a senior neuroradiologist with special interest in neurodegenerative disorders and NPH (OT); lumbar puncture for (f) cerebrospinal fluid neurodegeneration biomarkers and (g) clinical examination of gait and balance (Tinetti gait and balance test [26], Timed up and go test [27], 10 m walk and 360 right and left turns, single leg stand, Romberg’s test) before and after the procedure carried out by a neurophysiotherapist (CA). Final diagnoses were formulated by consensus in the multidisciplinary team meeting following tap test on the basis of aforementioned information. Standard treatment was a ventriculoperitoneal shunt inserted via a parietal approach using programmable valves set at the individual surgeon’s preference (medium to high pressure) followed by review within 3 months.

### Experimental protocol

A 13-camera 3-dimensional motion analysis system (Qualisys Track Manager v2023.2, Qualisys AB, Gothenburg, Sweden) was used to accurately track the movement of markers positioned on specific anatomical landmarks according to the full body Plug-in-Gait model. Participants were asked to walk the length of the gait lab (10 m) 2–4 times depending on ability and fatigue levels. While a range of gait support options was made available (e.g., assistance from partner/carer/researcher; use of a standardised walking aid such as a cane/stick; or walking unaided) for inclusion to the broader study examining gait, balance, and functional mobility tests, walking unaided was required for the 3D gait analysis component of this feasibility arm of the study. All participants except one were able to complete the 3D gait task without any assistance. Kistler Force plates (Kistler (Type 9281B), Winterthur, Switzerland) embedded in a bespoke floor-mounted walkway recorded ground reaction forces (100 Hz) under the feet. The full study protocol was designed to align to functional tests included as part of patients’ clinical work-up.

### Data processing

Gait data from the 10 m walk test were processed offline. Motion capture data were individually reconstructed, digitally labelled, and gap-filled in Qualisys Track Manager 2024 software. Marker trajectories were then exported for analysis in Visual3D biomechanics software (HAS Motion, Kingston, Canada) via c3d file format.

Using the pipeline function in Visual3D, joint coordinate (marker) and force data were smoothed using a 4th order Butterworth low-pass digital filter with cut-off frequencies of 6 and 25 Hz, based on a priori residual analysis [29]. Where possible, the automatic gait events function was used to determine instances of heel strike and toe-off for each gait cycle; correct identification of events was reliant on clean foot strikes on the force plate. In instances where force input was of poor quality resulting in inaccurate event detection, manual identification of gait events was used. Ground reaction forces were also obtained in Visual3D, normalised relative to body weight, for vertical, anterior-posterior, and medial-lateral directions. Where possible, lower limb joint moments were calculated using an inverse dynamics approach and were defined as external moments normalised to body mass. Gait cycles were normalised 0-100% from heel-strike to heel-strike for each side (left and right).

### Statistical analysis

Descriptive statistics are presented to summarise the participant demographics, clinical and radiological scores. IBM SPSS Statistics version 26 was used to analyse between-group demographics and spatiotemporal parameters (gait speed, cadence, cycle time, stride length and width). Skewness and kurtosis were considered. The Shapiro-Wilk test was used to assess normality, and homogeneity of variance was assessed using Levene’s test. Where appropriate, independent samples t-tests were used to examine the mean differences between groups (i.e., iNPH vs. HC) for normally distributed variables. Non-parametric equivalents (Mann-Whitney U) were used when the assumptions for parametric analyses were not met. Fisher’s Exact Test was used to assess male: female participant distribution across groups. The accepted level of significance was set at *p* < .05.

Patients with iNPH frequently placed multiple, consecutive foot strikes on a single force plate due to short step lengths, preventing the isolation of clean, single-step kinetics. As ground reaction forces (vertical, antero-posterior, and medio-lateral) are essential inputs to calculating internal joint moments, these metrics could not be computed and analysed.

Kinematic characteristics (rotations about each axis for the ankle, knee, hip, and pelvis) of gait were compared between groups using one-dimensional statistical parametric mapping (SPM) [30, 31]. Continuous waveform data for joint angles (flexion/extension, ab/adduction, and internal/external rotation) were normalised from 0 to 100% of the gait cycle (heel-strike to heel-strike). Regions of difference between group joint angle waveforms (i.e., iNPH vs. HC) that persisted for > 5% of the gait cycle [32]. were then identified using the spm1d MATLAB package (1D SPM independent samples t-test, spm1d.org v M.0.4.3, run on MATLAB v2020a). Between-group waveform differences (two-tailed, α = 0.05) are reported as *t*(df) and *p* values at the regions of significant difference.

The regions of difference between group joint angle waveforms identified by the SPM analysis were then used in predictive modelling. To determine which of these parameters best discriminated between participants with iNPH and healthy controls, a logistic regression model was trained and tested in Python (3.13.5,) using scikit-learn 1.7.2. Predictors were *z*-transformed prior to analysis to normalise scaling, and Synthetic Minority Oversampling Technique (SMOTE) [33] was used to address group imbalance during training.

Model development and evaluation employed repeated stratified five-fold cross-validation, with approximately 80% of the data used for training and 20% for out-of-fold (OOF) validation, shown in Supplementary Material Figure SM1. Performance was assessed using accuracy, precision, recall, F1 score, and area under the receiver-operating characteristic curve (ROC–AUC), with participant-level performance estimates obtained by aggregating OOF predictions across splits. Feature selection was embedded within the cross-validation framework; features were ranked using SHapley Additive exPlanations (SHAP), which provide locally accurate, model-agnostic estimates of feature contributions to model output [34]. Feature subsets of increasing size were evaluated using inner cross-validation to identify performance plateaus and assessed for stability [35]. Features selected in at least 50% of splits were retained as a consensus set, representing gait features that were robust to data perturbation and therefore most reproducibly associated with iNPH. For further details on model development, please see Supplementary Material – Statistical Model Methods.

## Results

Of the 25 participants recruited to the iNPH group, one patient was excluded from analyses due to an inability to complete the gait trials unassisted. Data from two additional participants (1 iNPH, 1 HC) could not be analysed due to marker occlusion and data quality issues leaving 23 iNPH and 18 control participants included in the analyses.

## Participant demographics

Participant demographics are summarised in Table 1. There were no significant differences found between groups for age, mass, or height. The proportion of males was higher in the iNPH group compared to the HC group (Fisher’s Exact Test, *p* < .001); the odds of being male were 12.6 times higher in the iNPH group than in the control group (OR = 12.6, 95% CI *[2.84*,* 55.84]*).

**Table 1:** Participant demographics

	iNPH (*n* = 23)	HC (*n* = 18)	*p*	Effect Size
Age (years)	77.26 ± 5.8	73.78 ± 6.1	0.071	0.584
Sex (M: F)	18:5	4:14	< 0.001	0.601
Mass (kg)	78.88 ± 13.9	74.52 ± 19.5	0.408	0.263
Height (m)	1.63 ± 0.07	1.62 ± 0.09	0.24	0.141

Notes – values reported as mean ± standard deviation; iNPH = idiopathic Normal Pressure Hydrocephalus; HC = Healthy Controls; effect sizes reported as Cohen’s d for age, mass, height and as Cramer’s V for sex

### iNPH patients’ clinical and radiological results

iNPH patients’ mean total score on the iNPH scale (gait, balance, and bladder) was 51 (SD 16.5), mean mRS was 2.39 (SD 1) and mean total score on ACE was 78 (SD 10.5). Eighteen of 23 patients (78%) were shunted (1 declined surgery, 1 was lost to follow-up, 1 died prior to surgery, and risks of intervention were deemed outweighing benefits by surgeon for 2 patients); 15 of those 18 patients (83%) were responders. Patients’ percentages of vascular risk factors were in keeping with the UK prevalences at the mean age of our cohort. No patient had clinically probable diagnostic features of another competing neurodegenerative or peripheral nervous system disease. Mean Evan’s index was 0.38 (SD 0.34), mean Radscale score was 9.26 (SD 1.63) and 21/23 had DESH. Full iNPH patient details can be found in Table 2.

**Table 2:** iNPH patient details

ID	sex	age	iNPHscale^a^	mRS^b^	ACE^c^	TT^d^	shunted^e^	responder^f^	DESH^g^	Evans^h^	Radscale^i^	HTN^j^	DM^k^	HCHOL^l^	CVATIA^m^	MIIHD^*n*^	Fazekas^o^	
1	male	89	29	3	77	pos	no	n/a	yes	0.39	11	yes	no	no	no	yes	3	
2	male	76	78	1	81	pos	yes	yes	yes	0.40	11	yes	no	yes	no	yes	3	
3	male	76	77	1	88	pos	yes	yes	yes	0.39	8	no	no	yes	no	yes	0	
4	female	77	35	3	86	pos	yes	yes	yes	0.31	8	yes	no	no	no	no	1	
5	male	84	25	4	74	pos	yes	no	yes	0.45	11	yes	no	yes	yes	no	2	
6	female	79	20	4	43	neg	yes	yes	yes	0.32	9	yes	yes	yes	no	no	3	
7	female	72	48	3	72	neg	no	n/a	no	0.36	6	no	no	yes	no	no	1	
8	male	76	43	3	72	pos	yes	yes	yes	0.50	11	yes	yes	yes	yes	yes	2	
9	female	68	50	2	77	pos	yes	yes	yes	0.35	9	yes	no	yes	no	no	1	
10	male	77	49	1	80	pos	yes	yes	yes	0.37	10	yes	no	yes	yes	no	2	
11	male	80	61	2	95	neg	yes	yes	yes	0.39	9	no	no	yes	no	no	1	
12	male	76	58	3	83	pos	yes	yes	yes	0.39	10	no	yes	yes	yes	no	2	
13	male	83	38	3	83	neg	deceased	n/a	yes	0.37	9	yes	no	yes	yes	yes	3	
14	female	74	55	1	89	pos	no	n/a	yes	0.31	9	yes	yes	no	no	no	2	
15	male	64	51	2	76	pos	yes	yes	yes	0.40	11	yes	no	no	no	yes	3	
16	male	83	83	2	78	pos	yes	no	yes	0.41	9	no	no	yes	no	no	2	
17	male	76	49	2	82	pos	yes	yes	yes	0.37	9	yes	yes	yes	yes	no	3	
18	male	70	59	2	73	pos	yes	no	yes	0.35	8	yes	no	yes	no	no	1	
19	male	78	34	3	66	pos	yes	yes	no	0.36	5	no	no	no	no	no	2	
20	male	79	51	2	82	pos	yes	yes	yes	0.38	11	no	yes	yes	no	no	1	
21	male	78	70	3	85	pos	no	n/a	yes	0.34	10	no	yes	yes	yes	yes	2	
22	male	88	55	2	65	pos	yes	yes	yes	0.40	8	yes	no	yes	no	no	1	
23	male	74	48	3	87	neg	yes	yes	yes	0.43	11	yes	no	yes	no	no	1	
Mean		77.2	51	2.39	78					0.38	9.26						1.83	
Median		77	50	2	80					0.38	9.00						2.00	
Standard Deviation		5.8	16.5	1	10.5					0.04	1.63						0.89	
Range		25	63	3	52					0.20	6.00						3.00	
Minimum		64	20	1	43					0.30	5.00						0.00	
Maximum		89	83	4	95					0.50	11.00						3.00	
Percentage^p^												65	30	80	30	30		

^a^Gothenburg iNPH scale, calculated from gait, balance and bladder domains, ^b^modified Rankin scale, ^c^score on the Addenbrooke’s cognitive examination III out of 100; ^d^Tap test in 18/23 positive; of 5 negative, 3 responded to surgery, 2 were not shunted; ^e^18/23 shunted, 1 declined surgery, 1 lost to follow-up, 1 died prior to surgery, in 2 risks of intervention were deemed outweighing benefits by surgeon; ^f^15/18 (83%) shunt responders, ^g^disproportionally enlarged subarachnoid space hydrocephalus, ^h^Evans’ index; ^i^Radscale; ^j^hypertension; ^k^diabetes mellitus; ^l^hypercholesterolemia; ^m^history of stroke or TIA; ^n^history of myocardial infarction or ischemic heart disease; ^o^ischemic burden on FLAIR according to Fazekas scale from 0 to 3; ^p^percentage of participants with history of HTN, DM, HCHOL, CVATIA, MIIHD

### Spatiotemporal gait parameters

Significant between-group differences were observed for all spatio-temporal parameters (Table 3). Compared to controls, participants with iNPH exhibited 25% longer cycle time (*p* < .001), 64% larger stride width (*p* < .001), 60% slower gait speed (*p* < .001) and 52% shorter stride length (*p* < .001). Effect sizes were large across all parameters, indicating robust group differences.

**Table 3:** Standard spatio-temporal gait parameters

			Mean ± SD	*p*	t	df	Effect Size
Gait speed (m/s)		iNPH (23)	0.48 ± 0.25	< 0.001	-10.351	39	3.257
		HC (18)	1.20 ± 0.18				
Cycle time		iNPH (23)	1.35 ± 0.23	< 0.001	5.104	28.66	1.458
		HC (18)	1.08 ± 0.08				
Stride length (m)		iNPH (23)	0.63 ± 0.30	< 0.001	-8.702	39	2.739
		HC (18)	1.31 ± 0.15				
Stride width (m)		iNPH (23)	0.18 ± 0.04	< 0.001	5.774	38	1.847
		HC (17)	0.11 ± 0.03				

Notes – Independent samples t-tests. Effect size reported as Cohen’s d. One control participant lacked complete stride width data

### 3D Kinematics

**Fig. 1:**
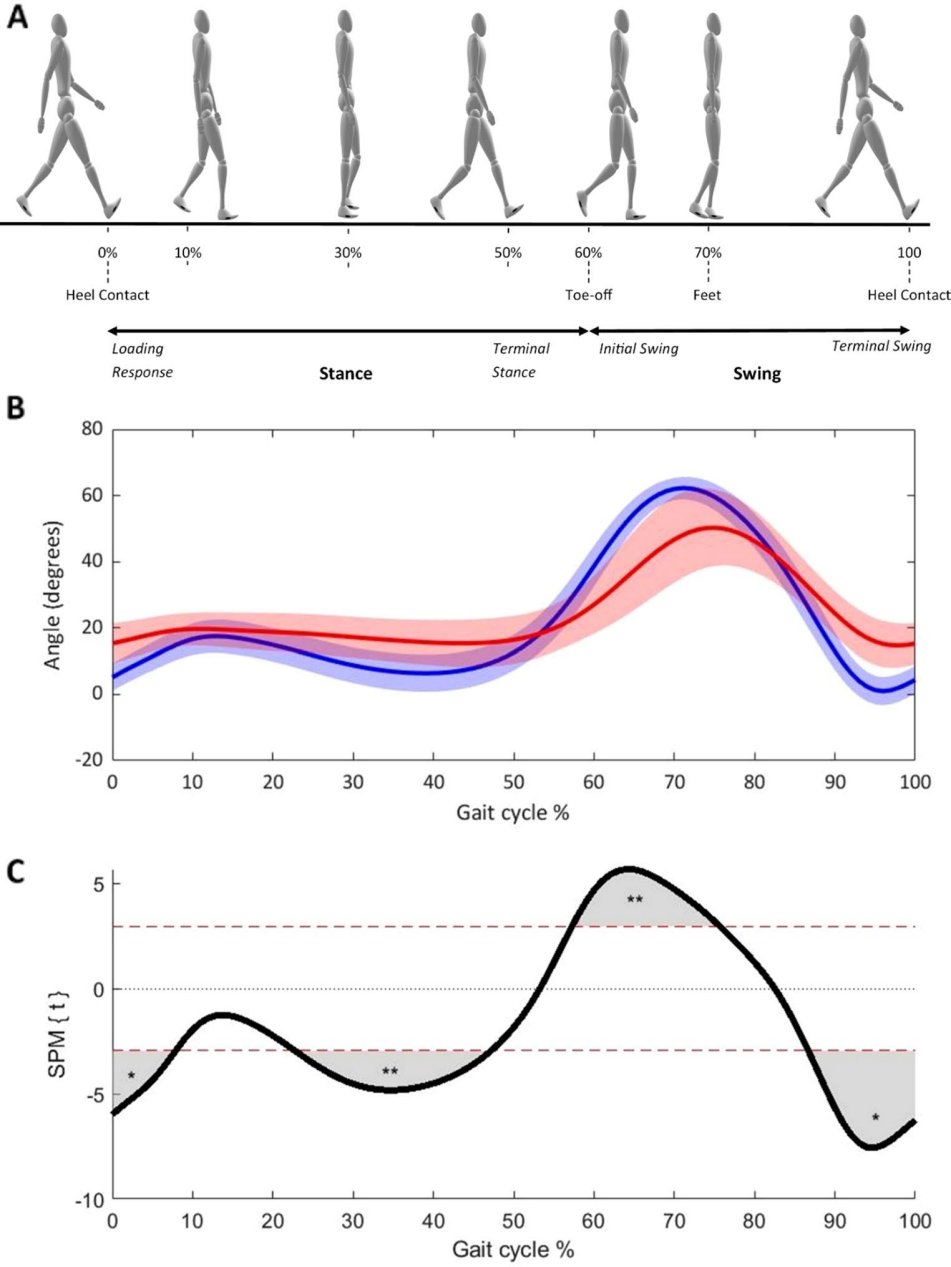
Knee joint sagittal angle kinematics and SPM hypothesis test results. (**A**) The walking human at various points of the gait cycle with typical approximations of stance and swing phases. (**B**) Mean (solid line) and standard deviations (light bands) of the knee joint angle in the sagittal plane (flexion-extension) across the gait cycle for HC (blue) and iNPH (red). (**C**) SPM hypothesis testing of the gait profiles. Regions of significant differences between groups are highlight in grey. *p < .05; **p < .01

Statistical parametric mapping revealed significant differences in lower-limb joint kinematics between individuals with iNPH and controls across all joints (pelvis, hip, knee, ankle). These differences were most pronounced in the sagittal and frontal planes of movement. Figure 1 illustrates sagittal plane knee joint angle kinematics and SPM hypothesis test results. Full SPM analysis figures for all joints in all planes can be found in Supplementary Material – Figure SM2.

At the pelvis, the iNPH group exhibited significantly reduced pelvic obliquity in early-mid stance (6–35% gait cycle), and during late stance, carrying through to mid-swing (57–84% gait cycle) (*t*(40) = 3.04, *p* < .001). Pelvic rotation was also significantly reduced in the iNPH group during mid-late stance phase (34–57% gait cycle) (*t*(40) = 2.98, *p* = .001), as well as in late-swing phase (81–100% gait cycle) (*t*(40) = 2.98, *p* = .004) transitioning into early stance (0–7%) (*t*(40) = 2.98, *p* = .034) of the next gait cycle. No differences were observed in the sagittal plane (i.e., pelvic tilt).

Participants with iNPH demonstrated reduced and delayed hip extension from mid stance through to toe-off, with significant differences observed between 25% and 64% of the gait cycle (*t*(40) = 2.55 *p* = .025). Hip adduction was also significantly reduced in the iNPH group during late swing (88–100% gait cycle) (*t*(40) = 2.85, *p* = .032), continuing into the next gait cycle until terminal stance (0–53% gait cycle) (*t*(40) = 2.85, *p* < .001). No differences between groups regarding internal/external rotation were observed for this joint.

In the knee, the iNPH group exhibited reduced and delayed flexion during terminal stance leading into swing phase (57%–76% of the gait cycle) (*t*(40) = 2.95, *p* = .007). The iNPH participants also displayed significantly less knee extension at terminal swing (87–100% gait cycle) (*t*(40) = 2.95, *p* = .018), continuing through to the next step (0–8% gait cycle) (*t*(40) = 2.95, *p* = .035), and again mid stance phase (*t*(40) = 2.95, *p* = .002) (Fig. 1). Significant differences between groups were not evident for the frontal (ab/adduction) and transverse (internal/external rotation) planes; though small-magnitude differences were noted in knee abduction, these occurred over less than 5% of the gait cycle continuously and were therefore considered to not be meaningful.

For the iNPH group, the ankle also remained in a dorsiflexed position for longer through to mid-late stance phase (36–49% gait cycle), not achieving plantarflexion (*t*(40) = 3.01, *p* = .013). This was followed by significantly reduced plantarflexion during terminal stance and reduced dorsiflexion during swing phase (55%–74% gait cycle) (*t*(40) = 3.01, *p* = .003). Ankle abduction was also significantly reduced during late stance phase for the iNPH group (47–68% gait cycle) (*t*(40) = 3.03, *p* = .001). No differences were observed for ankle internal/external rotation.

### Predictive modelling

**Fig. 2:**
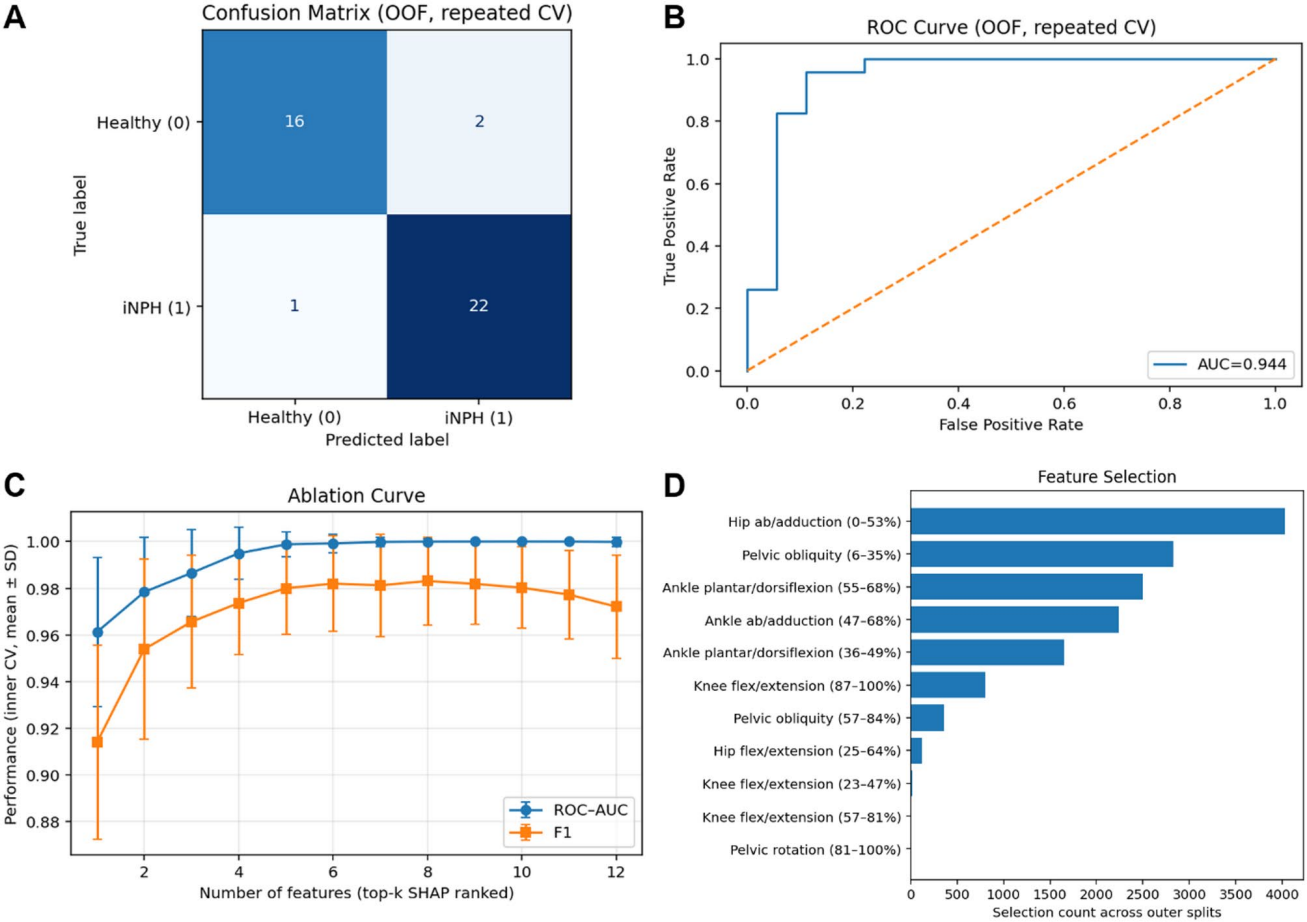
Classification performance of the nested cross-validation framework. (**A**) Confusion matrix showing out-of-fold predictions with 16/18 healthy controls and 22/23 iNPH patients correctly classified. (**B**) ROC curve demonstrating strong discriminative ability (AUC = 0.944) for the top 6 features (i.e., of panel D) guided by the SHAP importance. (**C**) Ablation analysis showing ROC-AUC increasing rapidly to 4 features, with ROC-AUC approaching 1.0 and F1 stabilising around 0.98 at 6 features. (**D**) Feature selection frequency across outer CV splits, with hip ab/adduction (0–53%) and pelvic obliquity (6–35%) emerging as the most consistently selected gait parameters (> 50% of the time). For features in panel (**D**), ‘%’ represents the %gait cycle regional differences, as identified in the SPM analysis. OOF: out-of-fold validation

**Fig. 3:**
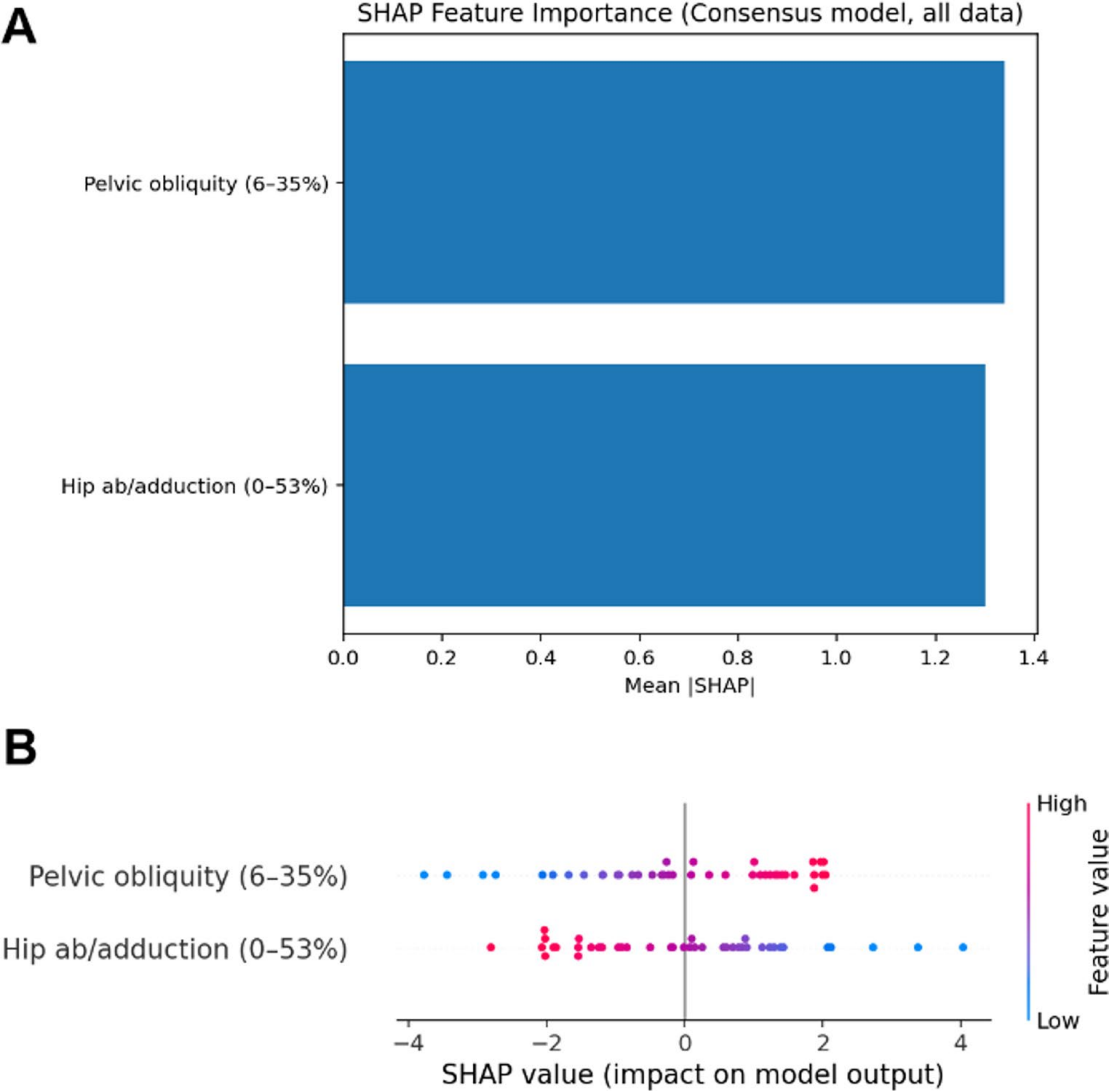
SHAP analysis of the consensus two-feature model for iNPH classification. (**A**) Mean absolute SHAP values showing equal contribution of pelvic obliquity and hip ab/adduction features. (**B**) Beeswarm plot revealing directional relationships: elevated pelvic obliquity (6–35% gait cycle) and reduced hip ab/adduction (0–53% gait cycle) are associated with iNPH classification. Feature values are colour-coded from low (blue) to high (red)

The logistic regression model achieved excellent discrimination between patients with iNPH and healthy controls (Fig. 2). The joint kinematic features initially identified in the SPM analysis were further refined using repeated five-fold cross-validation, feature ablation and stability selection. This identified that only two features, pelvic obliquity (6–35% of the gait cycle) and hip ab/adduction (0–53%) were required to achieve an AUC of 0.94 (95% CI 0.842, 1.000), with excellent sensitivity (0.96 [95% CI 0.857, 1.000]) and good specificity (0.89) and F1 (0.93 [95% CI 0.846, 1.000]) (Fig. 3).

## Discussion

We performed a detailed characterisation of gait function, assessing 3D joint kinematics in patients with iNPH and used these features to inform a binary logistic regression model for classification (iNPH vs. controls). As expected, standard spatiotemporal measures – slower walking speed, shorter stride length, wider step width – were altered in the iNPH group compared with controls. Joint kinematics of the pelvis, hip, knee, and ankle all demonstrated significant between-group differences, with excellent group separation reflected in the AUC analyses. These findings support the clinical utility of this protocol.

### Spatiotemporal dynamics

Our findings demonstrate significant impairments in spatiotemporal gait parameters in individuals with iNPH when compared to age-matched controls. The observed reductions in gait speed, stride length, and cadence, alongside increases in stride width and cycle time, are consistent with prior reports in iNPH populations (e.g., [10, 12, 19]) and align with the clinical characterisation of iNPH gait as short-stepped, broad-based, and slow. Importantly, the magnitude of between-group differences in our study was large, indicating these features are not merely age-related but disease-specific impairments.

While slower walking speed is a hallmark feature of aging [36], the combined presentation of slower walking speed with short stride length and increased stride width suggests compensatory adaptations to maintain postural stability during locomotion. In older adults, increased stride width is often interpreted as a stabilising strategy to counteract decreased dynamic balance [37]. Indeed, this is also seen in patients with diabetic peripheral neuropathy, where increased medio-lateral sway presents a challenge to dynamic balance [38]. However, in iNPH, such compensatory widening appears exaggerated when compared with healthy age-matched controls, potentially reflecting more profound supraspinal control deficits, as the underlying CSF dynamics may disrupt frontal-subcortical circuitry involved in gait regulation [7].

### Joint kinematics

Beyond basic spatiotemporal measures (e.g., gait speed, stride length/width, etc.), this study provides novel insights into the joint kinematics of iNPH gait. The multi-joint, multi-planar alterations observed, particularly in sagittal and frontal planes, indicate a complex disruption to motor control that affects both limb progression and postural stability throughout the gait cycle.

Reduced obliquity and rotation of the pelvis during stance and swing phases may reflect a diminished ability to shift weight dynamically, leading to a stiff or “en-bloc” strategy previously reported in iNPH [13]. This finding suggests truncal rigidity or impaired central coordination, possibly due to disruption of medial frontal and supplementary motor areas affected by ventricular enlargement [39].

The hip joint showed reduced extension during late stance and decreased abduction during late swing/early stance. Similar reductions in hip range of motion have been associated with increased fall risk in elderly populations [40], largely as a result of patterns that could restrict forward propulsion and lateral balance, emphasizing the clinical relevance of these impairments in functional independence and safety in iNPH.

Alterations in distal lower limb kinematics were evident across the knee and ankle joints. Participants with iNPH demonstrated reduced swing-phase flexion and decreased terminal stance extension, suggesting impaired limb clearance and reduced push-off, consistent with shuffling-type gait. These were accompanied by reduced ankle dorsiflexion during swing and attenuated plantarflexion during push-off, consistent with a loss of distal control during the gait cycle. Together, these present an increased risk of tripping [41]. While similar kinematic patterns have been observed in frail older adults with impaired neuromuscular coordination [42], the temporal shift and magnitude of these deficits in our cohort were more pronounced than would be expected from age alone. Further, these features may distinguish iNPH from Parkinsonian gait, which often shows reduced stride length but preserved knee flexion, particularly in early disease [43], though this would need to be confirmed through direct comparison.

Together, these features demonstrate strong ability to distinguish iNPH from age-related gait changes, with kinematic patterns consistent with central coordination failure. Furthermore, the use of statistical parametric mapping (SPM) adds methodological rigor, enabling time-continuous comparisons of joint angles and identifying specific phases of the gait cycle where deficits occur. These findings also point towards a potential role for interventions targeting intersegmental coordination and phase-specific gait mechanics, particularly those aimed at enhancing propulsion during late stance and improving limb clearance during swing. While the present study was not designed to evaluate therapeutic interventions, these biomechanical insights may provide a framework for more targeted, mechanism-informed physiotherapy approaches in individuals with iNPH.

### Predictive model viability

Considering the bee-swarm (Fig. 2, panel D), both selected features have equal effects on the model’s predictions, but in opposite directions. Higher values of pelvic obliquity (6–35% gait cycle), push model prediction towards the iNPH class, while lower hip ab/adduction values (0–53% gait cycle) push the model prediction towards the iNPH class. Given the high sensitivity and specificity, the model results suggest these selected features could simplify testing and should be investigated further for potential as a ‘digital biomarker’ of iNPH gait. However, the model should be further refined with a larger sample size, including greater variability across the iNPH scale, and validated against competing disorders, as the ablation analysis may require more features to distinguish between conditions.

### Clinical and diagnostic implications

The distinct combination of spatiotemporal deficits and multi-joint kinematic alterations provides a more comprehensive motor profile than traditional assessments or visual observation alone. From a diagnostic standpoint, gait abnormalities in iNPH often resemble those of other neurodegenerative or aging-related conditions, such as Parkinson’s disease (PD), vascular parkinsonism, or frailty syndromes [12, 44–46]. However, the detailed gait cycle phase-specific deficits identified here, such as delayed peak hip extension, attenuated knee flexion during swing, and blunted ankle push-off, suggest a central gait apraxia pattern more consistent with disrupted frontal-subcortical loops than with basal ganglia pathology, as seen in PD [11, 44, 45]. Indeed, structural and functional neuroimaging studies in iNPH have consistently demonstrated disruption of frontal–subcortical white matter pathways linking the supplementary motor area, basal ganglia, and brainstem locomotor regions [7, 39, 47]. This network-level disconnection is thought to impair the top-down modulation of gait initiation and interlimb coordination, yielding the broad-based, short-stepped, and shuffling phenotype observed here, and clinically recognised as a higher-level gait disorder [48].

In elderly populations, differentiating iNPH from age-related gait decline is an ongoing challenge, particularly because the more obvious features, such as reduced stride length and slower walking speed, occur in both groups. iNPH patients in this study were older than HCs at trend level. However, the extent and timing of the deficits observed here are unlikely to be explained by age alone. Age-related gait changes typically present with milder alterations in sagittal plane kinematics, while preserving overall joint coordination and step symmetry [49]. By contrast, the iNPH group in this study demonstrated a more global, phase-distributed dysfunction across joints, suggesting a higher-level deficit in motor planning and interlimb coordination.

We believe these distinctions are clinically meaningful and carry potential diagnostic weight. Accurate diagnosis remains a limiting factor in addressing the substantial undertreatment of iNPH, estimated to affect up to 60–80% of potential surgical candidates [3]. The reliance on coarse metrics of functional tests, such as 10MWT or TUG, in clinical practice may miss subtle but functionally relevant improvements following cerebrospinal fluid tap test (CSFTT) or shunting, particularly in borderline responders. As demonstrated here, 3D motion analysis may offer a more granular analysis of iNPH gait, to be considered to capture nuanced improvements or declines in motor coordination and timing that standard tests may overlook, particularly in patients with low severity iNPH. Integrating such detailed spatiotemporal data into CSFTT protocols may therefore enhance predictive accuracy and reduce false negatives.

### Limitations and future work

While strengths of this study include matched samples, diagnosis of iNPH in a specialist centre with surgical success rates comparable to those of established academic centres, and comprehensive and detailed assessment of gait function, some limitations should be acknowledged. First, the analysis of kinetic data was constrained by the technical limitations of force plate data collection, particularly due to the short, shuffling gait patterns common in individuals with iNPH, which often resulted in multiple foot strikes across each force plate. This limited our ability to assess ground reaction forces and calculate joint moments reliably. Force plates are also limited to ground reaction forces only, rather than pressure distributions across the feet (e.g., heel vs. forefoot loading). To address these, it is suggested that future studies investigate kinetics and/or pressure distributions via instrumented treadmills or pressure mat/insole systems. Additionally, despite the large effect sizes observed, the sample size was relatively small and may not be representative of the wider iNPH population. While epidemiological studies suggest a balanced distribution of sex in iNPH, our cohort was predominantly male which may limit generalizability. Further, the study included only individuals with a confirmed diagnosis of iNPH with moderately severe disease progression, without a comparison group consisting of patients with mimicking disorders such as Parkinson’s disease or progressive supranuclear palsy. Given the clinical overlap in gait presentation, future work should incorporate comparative cohorts to enhance diagnostic specificity. Future larger studies should also look to include patients with early diagnosis of iNPH (i.e., low severity) and account for comorbidities affecting gait as confounders in the statistical modelling to assess whether kinematics-based features are still able to differentiate. This would also allow for the investigation of the relationships between kinematic features and disease severity and progression.

Although 3D motion capture offers gold-standard precision, its use in clinical practice is currently limited by cost, infrastructure, and expertise. Future research should explore the feasibility of translating these biomechanical markers to more scalable technologies such as wearable inertial measurement units (IMUs) or markerless and/or AI-based motion analysis, which could facilitate broader clinical adoption, including in community or outpatient settings. Longitudinal studies are also warranted to investigate whether kinematic changes can track disease progression or response to interventions such as CSFTT or shunting, and whether early gait alterations might predict long-term outcomes, identifying which gait characteristics are most sensitive and specific for diagnosis and detection of change. The CSFTT can identify shunt responders when positive, but its negative predictive value is very low, leading to erroneous exclusion of patients from treatment. Probable reasons for this include insensitive assessment of improvements in gait performance lacking quantitative detail and qualitative aspects and limitations in timepoints of follow-up [50]. Development of a more sensitive gait assessment protocol following CSFTT, with a better quantification of improvements in both gait characteristics and performance is highly warranted since it would improve diagnostics and prediction, as well as follow-up assessments in iNPH.

## Conclusions

To our knowledge, this is the first study to provide a comprehensive characterisation of gait abnormalities in patients with idiopathic Normal Pressure Hydrocephalus (iNPH) by including both conventional spatiotemporal gait metrics and 3-dimensional (3D) kinematic analysis. It is also the first to report quantified gait parameters in this population using statistical parametric mapping. The results from this study not only broadly confirm and support previously documented gait impairments consistent with clinical presentation in terms of standard metrics but also present new findings to better understand these impairments, particularly with regards to sagittal and frontal plane 3D kinematics, including specific joint-level alterations across the pelvis, hip, knee, and ankle. Importantly, the features identified herein can also be used in logistic regression modelling to distinguish iNPH from HC with 96% sensitivity and 89% specificity. While these findings demonstratestrong potential for future use in differentiating between competing disorders, further validation in larger and clinically heterogeneous cohorts is required prior to clinical implementation.

## Supplementary Information

Below is the link to the electronic supplementary material.

Supplementary Material 1

Supplementary Material 2

Supplementary Material 3

## Data Availability

The datasets used and/or analysed during the current study are available from the corresponding author on reasonable request.

## Abbreviations

iNPH: idiopathic normal pressure hydrocephalus
3D: Three–dimensional
SPM: Statistical parametric mapping
10MWT: 10 min walk test
TUG: Timed up and go
NHS: National health services
mRS: Modified rankin scale
DESH: Disproportionally enlarged subarachnoid space hydrocephalus
HC: Healthy controls
SMOTE: Synthetic minority oversampling technique
SHAP: SHapley additive explanations
OR: Odds ratio
CI: Confidence interval
SD: Standard deviation
ACE: Addenbrooke’s cognitive exam
SM: Supplementary material
AUC: Area under the curve
ROC: Receiver operator curve
OOF: Out–of–fold
PD: Parkinson’s disease
CSFTT: Cerebrospinal fluid tap–test
