# Supplementary material for: Three-dimensional kinematic gait signatures of idiopathic normal pressure hydrocephalus: a biomechanical framework toward objective diagnosis
Source: Fluids Barriers CNS. 2026 May 22;23:75. doi: 10.1186/s12987-026-00813-6 (PMC13196002; doi:10.1186/s12987-026-00813-6)
Supplement: Supplementary file 1 — Supplementary Material 1 [file 12987_2026_813_MOESM1_ESM.docx]

***Statistical Modelling Methods***

Model development and evaluation were performed using a repeated stratified cross-validation, summarised in Figure SM1, below. We used 5-fold cross-validation with 1000 repeats, producing 5000 train-validation splits. For each split, 80% of the data was used for training and 20% held out for validation (out-of-fold (OOF))

All preprocessing steps, including missing-value imputation, feature standardisation, and class imbalance handling, were performed within the training data of each split only. Classification was performed using logistic regression, selected for its interpretability and suitability for small clinical datasets.

*Feature selection and ablation analysis*

Feature selection was embedded within the cross-validation procedure to prevent information leakage. Within each training split, feature importance was quantified using SHAP SHapley Additive exPlanations (Lundberg and Lee, 2017). Mean absolute SHAP values were aggregated across inner cross-validation folds to obtain a split-specific ranking of original gait features. To determine the appropriate set of features, ablation analysis was conducted by evaluating subsets of increasing size (top-1 to top-k features) ranked by SHAP importance. For each subset size, cross-validated performance was assessed using the area under the receiver-operating characteristic curve (ROC–AUC). The optimal subset size was defined as the smallest number of features that maximised AUC. The ablation curve is shown in Figure 2 in the manuscript.

*Feature stability and consensus selection*

Feature stability was assessed using stability-selection principles, whereby the frequency with which each feature was selected across resampling splits was recorded (Meinshausen and Bühlmann, 2010). Features selected in **at least 50% of splits** were retained as a consensus feature set, representing the most robust and reproducible gait characteristics associated with iNPH.

*Model interpretability*

For interpretability, a consensus model was trained on the full dataset using the stability-selected feature set. SHAP was used to visualise feature importance and the directionality of feature effects. These analyses were used solely for interpretation and were not employed for performance estimation.
