# Supplementary material for: Three-dimensional kinematic gait signatures of idiopathic normal pressure hydrocephalus: a biomechanical framework toward objective diagnosis
Source: Fluids Barriers CNS. 2026 May 22;23:75. doi: 10.1186/s12987-026-00813-6 (PMC13196002; doi:10.1186/s12987-026-00813-6)
Supplement: Supplementary file 2 — Supplementary Material 2 [file 12987_2026_813_MOESM2_ESM.docx]

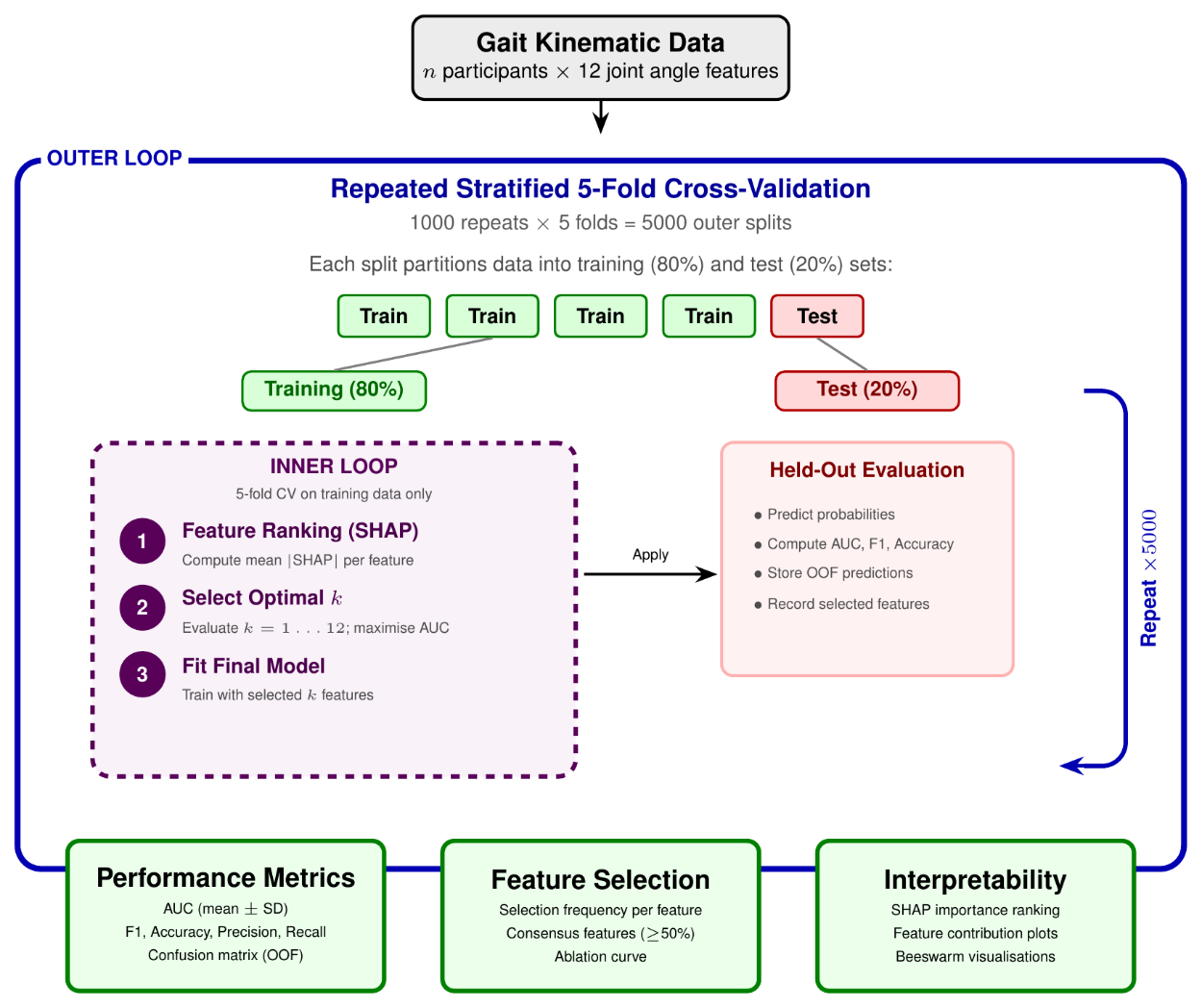


**Figure SM1 – Nested cross-validation framework for unbiased feature selection and classification performance estimation.** The outer loop uses repeated stratified 5-fold cross-validation (1000 repeats × 5 folds = 5000 splits) to generate independent training and test sets. Within each outer training fold, a separate 5-fold inner cross-validation ranks features by mean absolute SHAP value and selects the optimal feature subset size (k) by maximising AUC. This separation ensures feature selection decisions are made without exposure to outer test data, preventing information leakage. Out-of-fold predicted probabilities are averaged across repeats to obtain a single estimate. AUC (area under the receiver operating characteristic curve); OOF (out-of-fold); SHAP (SHapley Additive exPlanations).
