# Supplementary material for: Three-dimensional kinematic gait signatures of idiopathic normal pressure hydrocephalus: a biomechanical framework toward objective diagnosis
Source: Fluids Barriers CNS. 2026 May 22;23:75. doi: 10.1186/s12987-026-00813-6 (PMC13196002; doi:10.1186/s12987-026-00813-6)
Supplement: Supplementary file 3 — Supplementary Material 3 [file 12987_2026_813_MOESM3_ESM.docx]

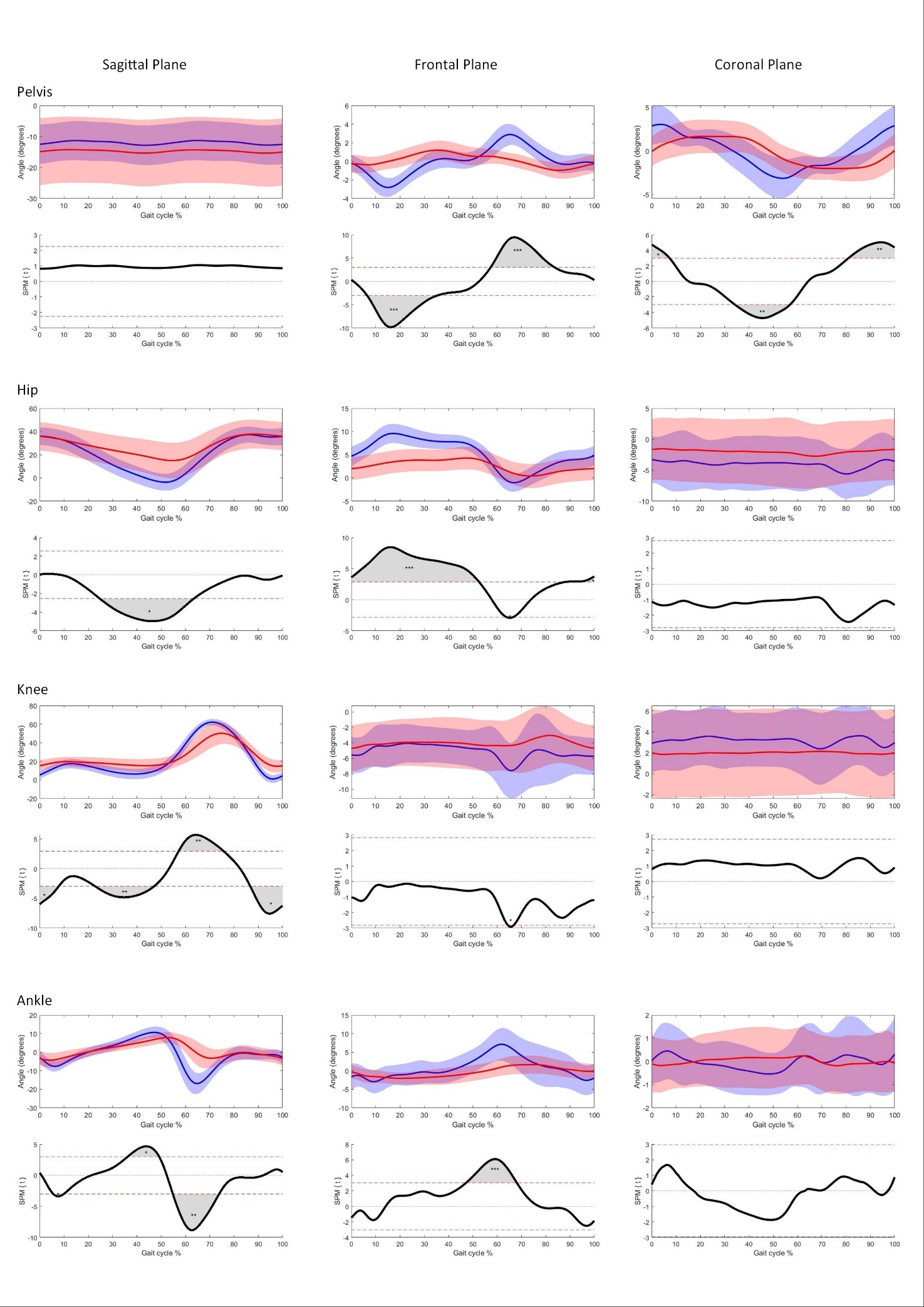


**Figure SM2 - Kinematics (joint angles) and SPM hypothesis test results for pelvis (top), hip (2nd from top), knee (2nd from bottom), and ankle (bottom).** For each joint, mean joint angle (solid line) and standard deviations (light bands) across the gait cycle are presented in the sagittal (left column), frontal (middle column), and coronal (right column) planes for HC (blue) and iNPH (red). (C) SPM hypothesis testing of the gait profiles. Regions of significant differences between groups are highlight in grey. *p<.05; **p<.01; ***p<.001
